# Evaluation of US State–Level Variation in Hypertensive Disorders of Pregnancy

**DOI:** 10.1001/jamanetworkopen.2020.18741

**Published:** 2020-10-01

**Authors:** Alexander J. Butwick, Maurice L. Druzin, Gary M. Shaw, Nan Guo

**Affiliations:** 1 Department of Anesthesiology, Perioperative and Pain Medicine, Stanford University School of Medicine, Stanford, California; 2 Department of Obstetrics and Gynecology, Stanford University School of Medicine, Stanford, California; 3 Division of Neonatal and Developmental Medicine, Department of Pediatrics, Stanford University School of Medicine, Stanford, California

## Abstract

**Importance:**

Hypertensive disorders of pregnancy are important causes of maternal and perinatal morbidity in the US. However, the extent of statewide variation in the prevalence of chronic hypertension, pregnancy-induced hypertension or preeclampsia, and eclampsia in the US remains unknown.

**Objective:**

To examine the extent of statewide variation in the prevalence of chronic hypertension, hypertensive disorders of pregnancy (including pregnancy-induced hypertension or preeclampsia), and eclampsia in the US.

**Design, Setting, and Participants:**

A cross-sectional study using 2017 US birth certificate data was conducted from September 1, 2019, to February 1, 2020. A population-based sample of 3 659 553 women with a live birth delivery was included.

**Main Outcomes and Measures:**

State-specific prevalence of chronic hypertension, hypertensive disorders of pregnancy, and eclampsia was assessed using multilevel multivariable logistic regression, with the median odds ratio (MOR) to evaluate statewide variation.

**Results:**

Of the 3 659 553 women, 185 932 women (5.1%) were younger than 20 years, 727 573 women (19.9%) were aged between 20 and 24 years, 1 069 647 women (29.2%) were aged between 25 and 29 years, 1 037 307 women (28.3%) were aged between 30 and 34 years, 523 607 women (14.3%) were aged between 35 and 39 years, and 115 487 women (3.2%) were 40 years or older. Most women had Medicaid (42.8%) or private insurance (49.4%). Hawaii had the lowest adjusted prevalence of chronic hypertension (1.0%; 95% CI, 0.9%-1.2%), and Alaska had the highest (3.4%; 95% CI, 3.0%-3.9%). Massachusetts had the lowest adjusted prevalence of hypertensive disorders of pregnancy (4.3%; 95% CI, 4.1%-4.6%), and Louisiana had the highest (9.3%; 95% CI, 8.9%-9.8%). Delaware had the lowest adjusted prevalence of eclampsia (0.03%; 95% CI, 0.01%-0.09%), and Hawaii had the highest (2.8%; 95% CI, 2.2%-3.4%). The degree of statewide variation was high for eclampsia (MOR, 2.36; 95% CI, 1.88-2.82), indicating that the median odds of eclampsia were 2.4-fold higher if the same woman delivered in a US state with a higher vs lower prevalence of eclampsia. Modest variation between states was observed for chronic hypertension (MOR, 1.27; 95% CI, 1.20-1.33) and hypertensive disorders of pregnancy (MOR, 1.17; 95% CI, 1.13-1.21).

**Conclusions and Relevance:**

The findings of this study suggest that after accounting for patient-level and state-level variables, substantial state-level variation exists in the prevalence of eclampsia. These data can inform future public-health inquiries to identify reasons for the eclampsia variability.

## Key Points

**Question:**

Does the prevalence of chronic hypertension, hypertensive disorders of pregnancy, and eclampsia vary across the US by state?

**Findings:**

In this cross-sectional study of 3 659 553 women who had live births in the US in 2017, the median odds of eclampsia were 2.4-fold higher if the same woman delivered in a state with a higher vs lower prevalence of eclampsia. The median odds ratios were substantially lower for chronic hypertension and hypertensive disorders of pregnancy.

**Meaning:**

The findings of this study suggest that substantial variation among states exists in the prevalence of eclampsia across the US, despite controlling for multiple patient characteristics.

## Teaser

This cross-sectional study examines statewide variations in the prevalence of hypertensive disorders in pregnant women across the US.

## Introduction

Hypertensive disorders that impact pregnant women, which include chronic hypertension, pregnancy-induced hypertension or preeclampsia, and eclampsia, are important causes of maternal death. Between 2011 and 2014, hypertensive disorders accounted for 7.1% of all US maternal deaths.^1^ Hypertensive disorders, especially severe preeclampsia and hemolysis, elevated liver enzyme levels, and low platelet count syndrome, are associated with adverse perinatal and maternal outcomes, including preterm delivery, intrauterine growth retardation, placental abruption, stroke, renal failure, and long-term cardiovascular morbidity.^2,3^

In population-based studies using administrative data, the reported US prevalence of hypertensive disorders was 1.7% to 1.8% for chronic hypertension, 3.0% to 3.8% for pregnancy-induced hypertension, 3.0% to 3.4% for preeclampsia, and 0.08% for eclampsia.^4,5,6,7,8^ However, the extent of regional variation for these disorders is unknown. Identifying such variation has important implications for state and national health policy,^9^ national guidelines,^10,11^ and etiologic research.

The main objective of this study was to examine the extent of statewide variation in the prevalence of chronic hypertension, pregnancy-induced hypertension or preeclampsia, and eclampsia in the US. We hypothesized that, after adjustment for patient-level and state-level factors, the prevalence of chronic hypertension, pregnancy-induced hypertension or preeclampsia, and eclampsia varies among states across the US. To test this hypothesis, we conducted a retrospective cross-sectional analysis of approximately 3.6 million women who had a live birth in the US in 2017.

## Methods

We performed a population-based cross-sectional study using 2017 US birth certificate data. The study was conducted from September 1, 2019, to February 1, 2020. Because the birth certificate data we used were publicly available and deidentified, we obtained an exemption of review from the Stanford University institutional review board. We followed Strengthening the Reporting of Observational Studies in Epidemiology (STROBE) guideline for cross-sectional studies. We sourced birth certificate data for 100% of US live births in all 50 states and the District of Columbia. Information on US birth certificates follows the 2003 revised US Standard Certificate of Live Birth format and comprises demographic, medical, obstetric, and neonatal data.^12^ On the revised birth certificate, the National Center for Health Statistics describes hypertensive disorders as prepregnancy (or chronic), gestational (which includes pregnancy-induced hypertension or preeclampsia), and eclampsia; definitions and key words for these birth certificate variables are published by the US National Center for Health Statistics.^13^ To avoid ambiguity in terminology, we have relabeled *gestational hypertension* as *hypertensive disorders of pregnancy*, which is in line with definitions used by the American College of Obstetricians and Gynecologists.^11^ Based on specifications for reporting items on the birth certificate, birth coders can report chronic hypertension or hypertensive disorders of pregnancy individually but not together.^12^ Therefore, this mutual exclusivity precluded us from identifying women with chronic hypertension and preeclampsia (ie, superimposed preeclampsia). Based on data from 3 states, the positive predictive value for hypertension disorders reported on the revised birth certificate is high (99%).^14^

We initially identified women who underwent a live birth in 2017. We excluded women who had missing data for chronic hypertension, hypertensive disorders of pregnancy, and eclampsia; gestational age at delivery; and any patient-level factor. Frequencies of missingness for each type of hypertensive disorder and patient factors are presented in eTable 1 in the Supplement. Given the low rates of missingness for these variables, we performed complete case analyses. Other exclusion criteria included women with gestational age at delivery reported as less than 20 weeks or more than 45 weeks.

To examine state-level variation in the prevalence of chronic hypertension, hypertensive disorders of pregnancy, and eclampsia, we created 3 analytic samples for each type of hypertensive disorder. Because South Carolina and Tennessee did not report eclampsia, births in these states were excluded from the analytic sample for eclampsia.

### Patient-Level Factors

The following patient-level factors were selected for inclusion in multivariable models: maternal age in years (<20, 20-24, 25-29, 30-34, 35-39, and >40 years) race/ethnicity (non-Hispanic White, non-Hispanic Black, non-Hispanic other, non-Hispanic Asian, and Hispanic), educational level (high school or less, college or associated bachelor’s degree, and master’s or doctorate degree), insurance (private insurance, Medicaid, self-pay, or other), prepregnancy body mass index using the World Health Organization categories,^15^ smoking history before pregnancy (presence or absence), prepregnancy diabetes (presence or absence), and number of prior live births (0 or ≥1). Race and ethnicity were examined as disparities exist in all hypertensive disorders^16^ and population distributions in states vary by race and ethnicity. Race and ethnicity are categorized by the National Center for Health Statistics in the birth certificate data files. In the models for hypertensive disorders of pregnancy and eclampsia, we also included gestational diabetes (presence or absence), plurality (single, twin, or triplet, or higher-order pregnancy) and trimester when women initiated prenatal care (first, second, or third trimester) as additional covariates. Because these variables are pregnancy related and cannot precede a prepregnancy diagnosis of chronic hypertension, they were not included in the chronic hypertension models. Women with missing details for prenatal care were also excluded from the analytic samples for pregnancy-related disorders and eclampsia.

### Statistical Analysis

We performed multilevel logistic regression using the GLIMMIX procedure in SAS version 9.4 (SAS Institute), with maximum likelihood estimation based on the Laplace approximation. We fit 3 regression models as follows: a null model that included state as a random effect (model 1), a model that included patient-level factors (model 2), and a model that included patient-level and state-level factors (model 3).

State-level factors in model 3 included 2017 median household income taken from the American Community Survey^17^ and percentage of families with a household income below the poverty level obtained from the American Community Survey for 2013 to 2017.^18^ We also included data for the number of general practitioners per 1000 deliveries (included only in the model for chronic hypertension) and number of obstetrician-gynecologists per 1000 deliveries (included in models for hypertensive disorders of pregnancy and eclampsia) using 2018 data from the US Bureau of Labor Statistics.^19^ We did not include data for both physician specialties in models for all hypertensive disorders because we assumed that general practitioners may manage care for women with chronic hypertension and obstetricians may manage care for women with hypertensive disorders of pregnancy.

In each model, we quantified the between-state variation using random-effects variance by computing the median odds ratio (MOR).^20,21,22^ In this study, the MOR indicates the extent to which an individual’s probability of hypertensive disease is determined by the state and is comparable to an OR used for patient-level factors. An MOR of 1.0 implies that there are no differences between states in the odds of a woman developing a hypertensive disorder. An MOR greater than 1 implies state-level variation in the individual odds of developing a hypertensive disorder: the larger the OR, the stronger the variation. We calculated 95% CIs for each MOR value. Detailed information about the quantification of state-level adjusted prevalence and the MOR are presented in the eMethods in the Supplement. Prevalence data for individual states are presented as caterpillar plots and heat maps.

We performed several prespecified analyses. First, we conducted stratified analyses for the prevalence of eclampsia for women with and without hypertensive disorders of pregnancy. Because chronic hypertension and hypertensive disorders of pregnancy cannot both be checked on the 2003 revised birth certificate, we could not perform stratified analyses for the prevalence of preeclampsia for women with and without chronic hypertension. To obtain finer resolution of prevalence, we also calculated crude county-level prevalence of chronic hypertension, hypertensive disorders of pregnancy, and eclampsia for counties that had equal to or more than 100 deliveries in 2017.

## Results

We identified 3 855 500 US live births in 2017. Complete data were available on 3 659 553 women (Table 1); 185 932 women (5.1%) were younger than 20 years, 727 573 women (19.9%) were aged between 20 and 24 years, 1 069 647 women (29.2%) were aged between 25 and 29 years, 1 037 307 women (28.3%) were aged between 30 and 34 years, 523 607 women (14.3%) were aged between 35 and 39 years, and 115 487 women (3.2%) were 40 years or older. Most women had Medicaid (42.8%) or private insurance (49.4%). A flow diagram depicting women who met exclusion criteria and numbers in our final analytic samples is presented in eFigure 1 in the Supplement. The final analytic samples for assessing state-level variation included 3 659 553 deliveries for chronic hypertension, 3 588 122 deliveries for hypertensive disorders of pregnancy, and 3 461 192 deliveries for eclampsia. In these samples, the prevalence was 1.9% (95% CI, 1.9%-1.9%) for chronic hypertension, 6.5% (95% CI, 6.4%-6.5%) for hypertensive disorders of pregnancy, and 0.3% (95% CI, 0.3%-0.3%) for eclampsia; the prevalence for any hypertensive disorder was 8.6% (95% CI, 8.5%-8.6%).

**Table 1.** Characteristics of Women With Chronic Hypertension, Hypertensive Disorders of Pregnancy, and Eclampsia

Variable	Women, No. (%)						
	Live birth (N=3 659 553)	Chronic hypertension		Hypertensive disorders of pregnancy		Eclampsia^a^	
		Yes (n=69 158)	No (n=3 590 395)	Yes (n=232 171)	No (n=3 355 951)	Yes (n=9737)	No (n=3 451 455)
Maternal age, y							
<20	185 932 (5.1)	1328 (1.9)	184 604 (5.1)	12 792 (5.5)	168 929 (5.0)	624 (6.4)	172 652 (5.0)
20-24	727 573 (19.9)	8122 (11.7)	719 451 (20.0)	46 085 (19.8)	665 474 (19.8)	2004 (20.6)	678 045 (19.6)
25-29	10 69647 (29.2)	16 476 (23.8)	1 053 171 (29.3)	65 421 (28.2)	983 324 (29.3)	2642 (27.1)	1 006 468 (29.2)
30-34	1 037 307 (28.3)	21 446 (31.0)	101 5861 (28.3)	63 151 (27.2)	955 387 (28.5)	2549 (26.2)	984 982 (28.5)
35-39	523 607 (14.3)	16 280 (23.5)	507 327 (14.1)	35 099 (15.1)	479 091 (14.3)	1469 (15.1)	499 002 (14.5)
≥40	115 487 (3.2)	5506 (8.0)	109 981 (3.1)	9623 (4.1)	103 746 (3.1)	449 (4.6)	110 306 (3.2)
Maternal race/ethnicity							
Non-Hispanic							
White	1 921 162 (52.5)	33 669 (48.7)	188 7493 (52.6)	130 784 (56.3)	175 8125 (52.4)	4918 (50.5)	1 803 932 (52.3)
Black	529 163 (14.5)	20 255 (29.3)	508 908 (14.2)	40 153 (17.3)	472 411 (14.1)	2088 (21.4)	480 123 (13.9)
Other	115 944 (3.2)	2481 (3.6)	113 463 (3.2)	7937 (3.4)	105 529 (3.1)	587 (6.0)	110 061 (3.2)
Asian	238 147 (6.5)	2345 (3.4)	235 802 (6.6)	8635 (3.7)	225 594 (6.7)	483 (5.0)	231 062 (6.7)
Hispanic	855 137 (23.4)	10 408 (15.0)	844 729 (23.5)	44 662 (19.2)	794 292 (23.7)	1661 (17.1)	826 277 (23.9)
Insurance							
Medicaid	1 564 501 (42.8)	32 481 (47.0)	1 532 020 (42.7)	97 492 (42.0)	143 2004 (42.7)	4234 (43.5)	1462194 (42.4)
Private	1 809 020 (49.4)	32 413 (46.9)	1 776 607 (49.5)	121 051 (52.1)	165 9462 (49.4)	4719 (48.5)	1 721 175 (49.9)
Self-pay	145 818 (4.0)	1444 (2.1)	144 374 (4.0)	5474 (2.4)	136 311 (4.1)	300 (3.1)	138 484 (4.0)
Other	140 214 (3.8)	2820 (4.1)	137 394 (3.8)	8154 (3.5)	128 174 (3.8)	486 (5.0)	129 602 (3.8)
Maternal educational level							
High school or lower	1 409 083 (38.5)	26 334 (38.1)	1 382 749 (38.5)	85 548 (36.9)	129 1276 (38.5)	3968 (40.7)	1 322 305 (38.3)
College/associate /bachelor’s degree	1 807 965 (49.4)	35 845 (51.8)	177 2120 (49.4)	121 074 (52.1)	165 4608 (49.3)	4818 (49.5)	1706589 (49.4)
Master’s/doctorate degree	442 505 (12.1)	6979 (10.1)	435 526 (12.1)	25 549 (11.0)	410 067 (12.2)	951 (9.8)	422 561 (12.2)
Prepregnancy smoking	330 157 (9.0)	8446 (12.2)	321 711 (9.0)	22 413 (9.7)	300 582 (9.0)	959 (9.8)	302 800 (8.8)
Prepregnancy BMI							
Normal or underweight (<18.5 to 24.9)	1707077 (46.6)	11 767 (17.0)	169 5310 (47.2)	66 888 (28.8)	1 605 760 (47.8)	2973 (30.5)	1 612 714 (46.7)
Overweight (25.0-29.9)	960 509 (26.2)	14 509 (21.0)	946 000 (26.3)	60 832 (26.2)	881 066 (26.3)	2505 (25.7)	907 627 (26.3)
Obese (≥30)							
Class I	538 543 (14.7)	14 991 (21.7)	523 552 (14.6)	46 937 (20.2)	481 396 (14.3)	1934 (19.9)	507 153 (14.7)
Class II	264 564 (7.2)	118 99 (17.2)	252 665 (7.0)	29 662 (12.8)	230 010 (6.9)	1219 (12.5)	247 892 (7.2)
Class III	188 860 (5.2)	15 992 (23.1)	172 868 (4.8)	27 852 (12.0)	157 719 (4.7)	1106 (11.4)	176 069 (5.1)
Prepregnancy diabetes	33 423 (0.9)	5452 (7.9)	27 971 (0.8)	5127 (2.2)	27 590 (0.8)	309 (3.2)	30 931 (0.9)
Gestational diabetes	233 431 (6.4)	NA	NA	27521 (11.9)	202 314 (6.0)	1080 (11.1)	220 922 (6.4)
Prior live birth							
None	1 390 906 (38.0)	23 329 (33.7)	1 367 577 (38.1)	115 613 (49.8)	124 8109 (37.2)	4768 (49.0)	1 310 956 (38.0)
≥1	226 864 (62.0)	45 829 (66.3)	2 222 818 (61.9)	116 558 (50.2)	210 7842 (62.8)	4969 (51.0)	2 140 499 (62.0)
Plurality, pregnancy							
Singleton	3535150 (96.6)	NA	NA	216 673 (93.3)	3 250 156 (96.8)	8918 (91.6)	3 335 246 (96.6)
Twin	120 738 (3.3)	NA	NA	14 954 (6.4)	102 788 (3.1)	784 (8.0)	112 827 (3.3)
Triplet or higher-order	3665 (0.1)	NA	NA	544 (0.2)	3007 (0.1)	35 (0.4)	3382 (0.1)
Prenatal care initiated, trimester							
Missing	71 431 (2.0)	NA	NA	NA	NA	NA	NA
First	2 788 901 (76.2)	NA	NA	184 982 (79.7)	2 603 919 (77.6)	7488 (76.9)	2 686 748 (77.8)
Second	588 112 (16.1)	NA	NA	35 556 (15.3)	552 556 (16.5)	1661 (17.1)	563 013 (16.3)
Third	157 017 (4.3)	NA	NA	8723 (3.7)	148 294 (4.4)	389 (4.0)	150 855 (4.4)

Abbreviations: BMI, body mass index (calculated as weight in kilograms divided by height in meters squared); NA, not applicable.

^a^ South Dakota and Tennessee were excluded because they did not report eclampsia.

Table 1 presents the demographic characteristics of women in each analytic sample. eTable 2 in the Supplement presents adjusted ORs from multilevel models adjusting for patient-level factors. Maternal factors associated with all 3 hypertensive disorders were age 35 years or older, non-Hispanic Black race, overweight and all obesity classes, and prepregnancy diabetes. Medicaid was associated with chronic hypertension and eclampsia, while self-pay was inversely associated with chronic hypertension and hypertensive disorders of pregnancy. Gestational diabetes, twin pregnancy, and triplet or higher-order pregnancy were associated with hypertensive disorders of pregnancy and eclampsia. No prenatal care was associated with eclampsia.

### Prevalence of Hypertensive Disorders

**Figure 1.**
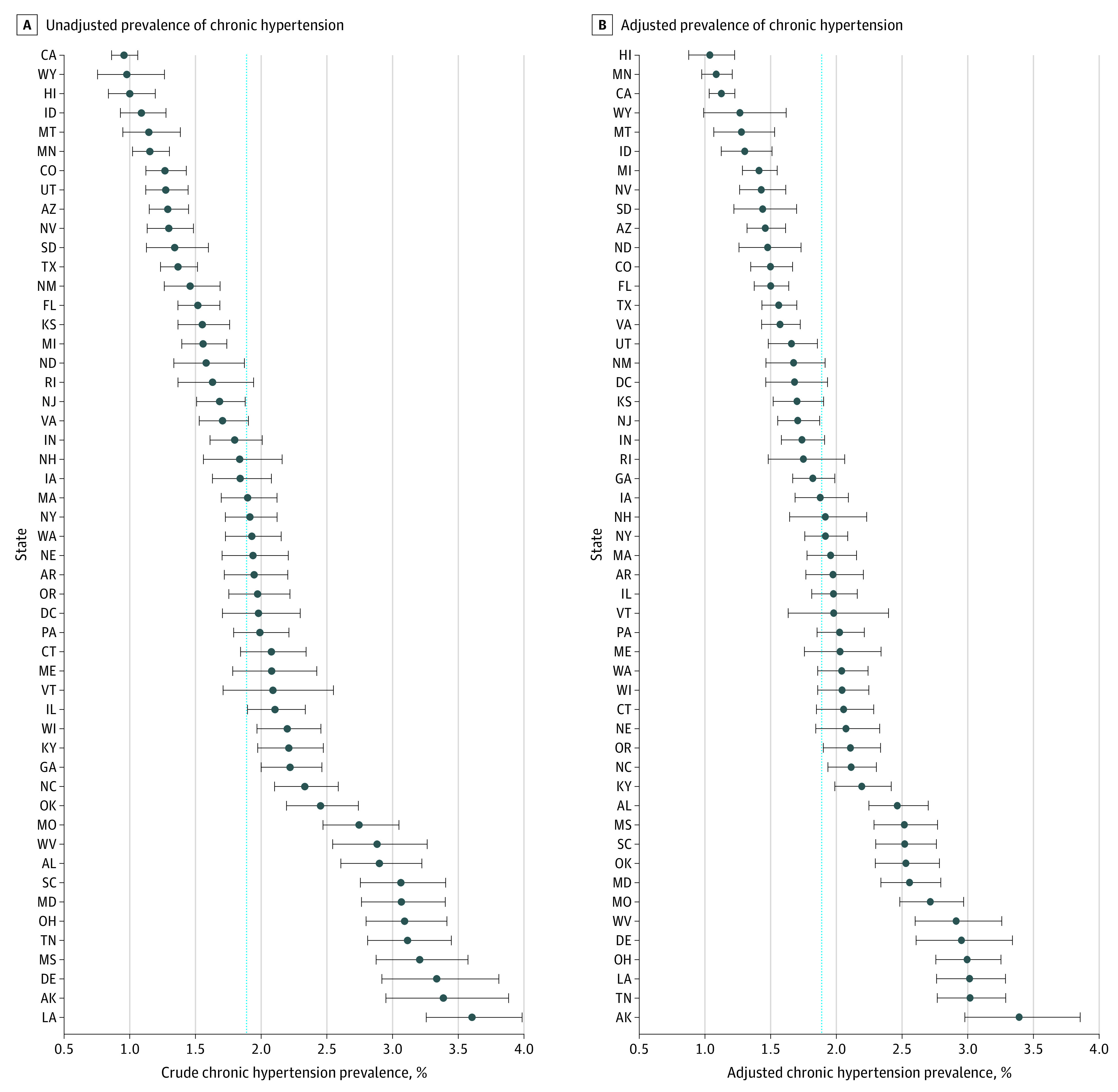
Prevalence of Chronic Hypertension The circles indicate the mean prevalence. The whisker bars indicate 95% CIs. Dashed horizontal line indicates the overall mean prevalence for all states.

**Figure 2.**
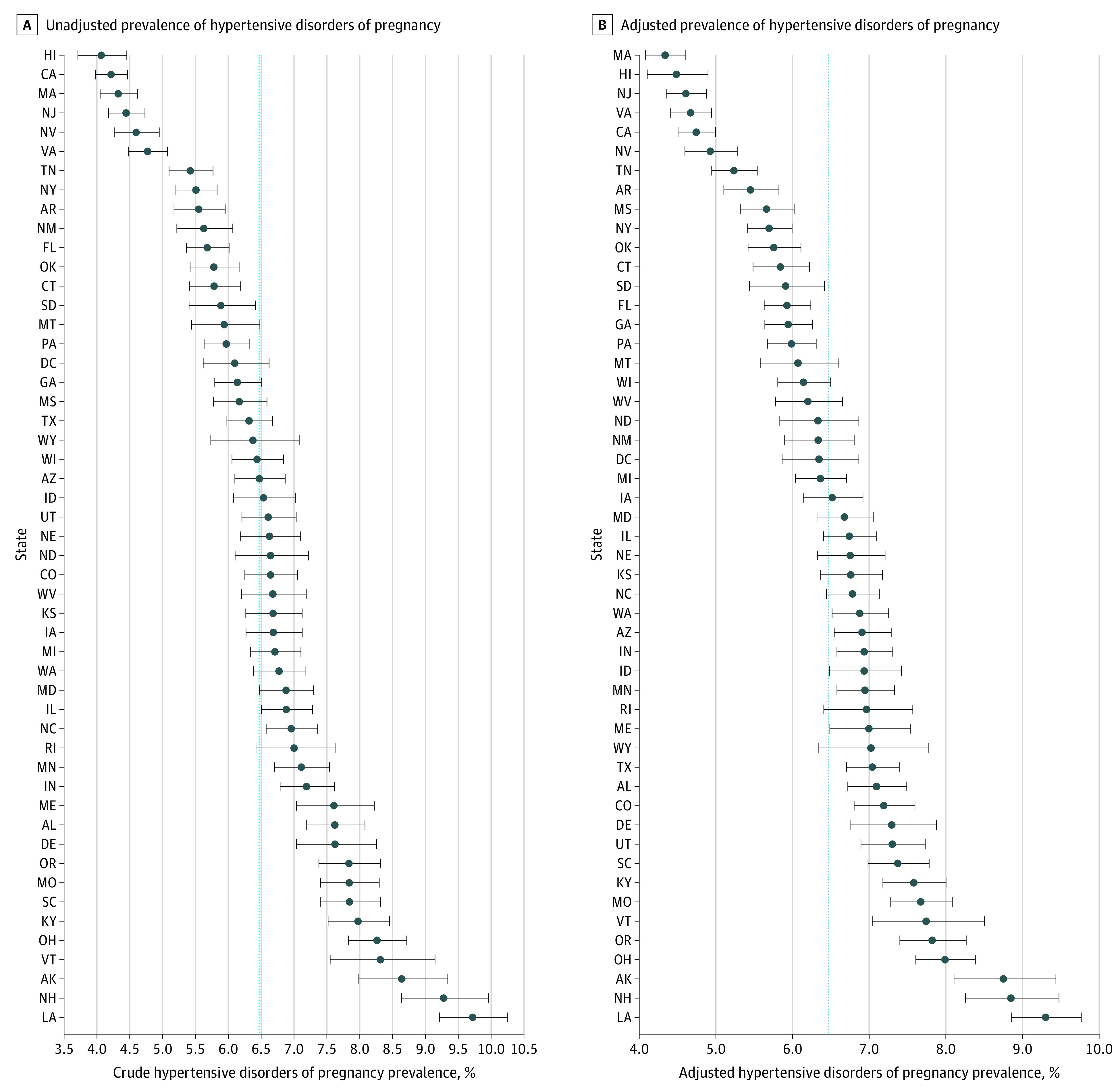
Prevalence of Hypertensive Disorders of Pregnancy The circles indicate the mean prevalence. The whisker bars indicate 95% CIs. Dashed horizontal line indicates the overall mean prevalence for all states.

**Figure 3.**
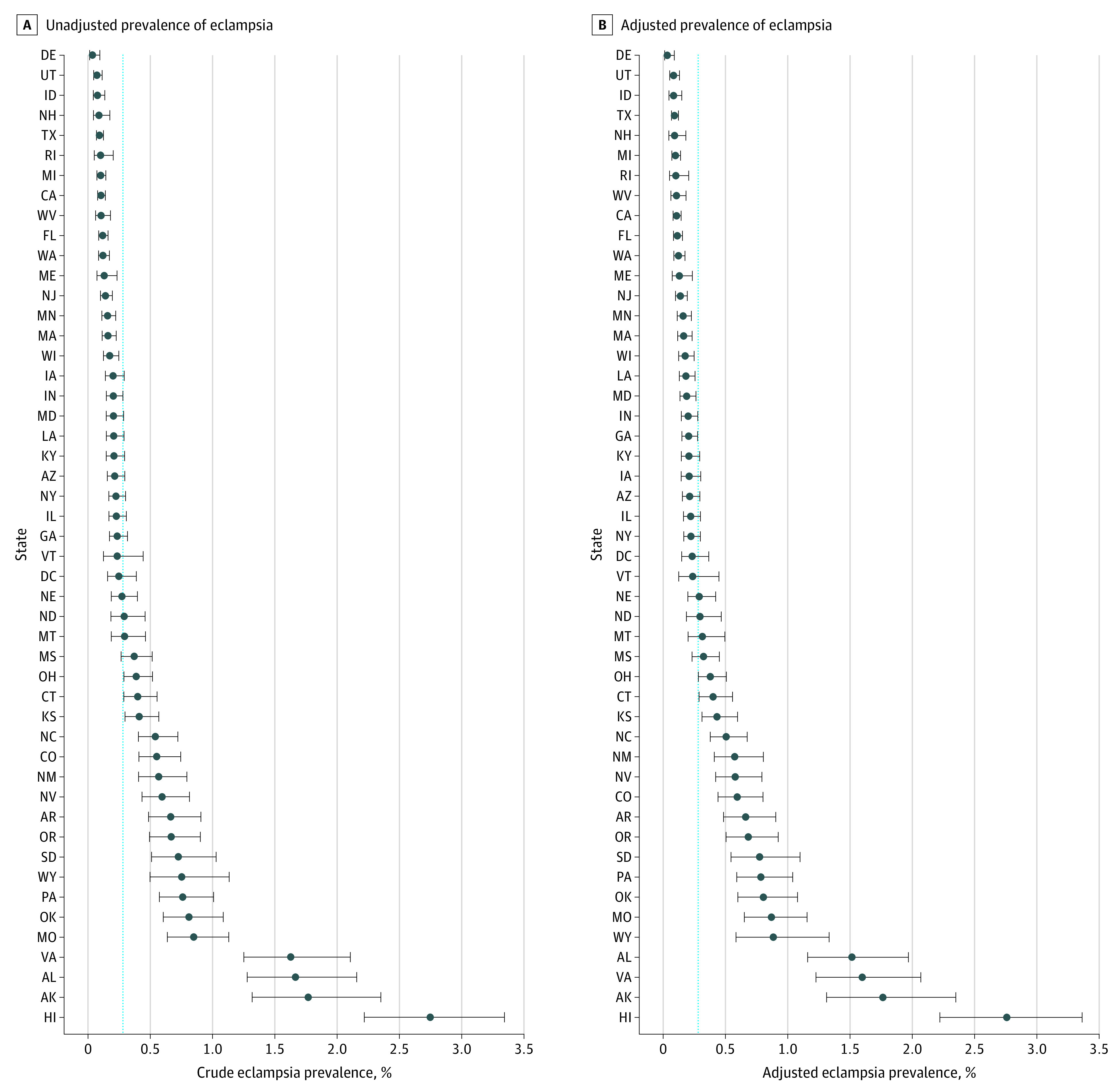
Prevalence of Eclampsia The circles indicate the mean prevalence. The whisker bars indicate 95% CIs. Dashed horizontal line indicates the overall mean prevalence for all states. South Carolina and Tennessee did not report eclampsia; therefore, births in these states were excluded from the analytic sample.

Caterpillar plots are presented for the unadjusted and adjusted prevalence of chronic hypertension (Figure 1), hypertensive disorders of pregnancy (Figure 2), and eclampsia (Figure 3). eFigures 2, 3, and 4 in the Supplement show heat maps for the adjusted state-level prevalence. The adjusted chronic hypertension prevalence ranged from 1.0% (95% CI, 0.9%-1.2%) in Hawaii to 3.4% (95% CI, 3.0%-3.9%) in Alaska. The adjusted hypertensive disorders of pregnancy prevalence ranged from 4.3% (95% CI, 4.1%-4.6%) in Massachusetts to 9.3% (95% CI, 8.9%-9.8%) in Louisiana. The adjusted eclampsia prevalence ranged from 0.03% (95% CI, 0.01%-0.09%) in Delaware to 2.8% (95% CI, 2.2%-3.4%) in Hawaii. Three other states (Alabama, Virginia, and Alaska) had an eclampsia prevalence greater than 1% (eTable 3 in the Supplement). Alaska and Missouri were among the 10 states with the highest adjusted prevalence for each hypertensive disorder (eTable 3 in the Supplement).

Table 2 presents the MORs of the unadjusted and adjusted models for the 3 hypertensive disorders. In model 1, the point estimates for the MORs were greater than 1 in all analytic samples. After sequential adjustment for patient-level factors (model 2) and then state-level factors (model 3), there was a slight decrease in the MORs in all analytic samples. The degree of statewide variation was high for eclampsia (MOR, 2.54; 95% CI, 1.88-2.82), indicating that the median odds of eclampsia were 2.4-fold higher if the same woman delivered in a US state with a higher vs lower prevalence of eclampsia. Modest variation between states was observed for chronic hypertension (MOR, 1.27; 95% CI, 1.20-1.33) and hypertensive disorders of pregnancy (MOR, 1.17; 95% CI, 1.13-1.21).

**Table 2.** State-Level Variation in Chronic Hypertension, Hypertensive Disorders of Pregnancy, and Eclampsia

Variable	Chronic hypertension			Hypertensive disorders of pregnancy			Eclampsia		
	Model 1^a^	Model 2^b^	Model 3^c^	Model 1^a^	Model 2^d^	Model 3^e^	Model 1^a^	Model 2^d^	Model 3^e^
State-level variance (SE)	0.131 (0.027)	0.085 (0.017)	0.063 (0.013)	0.043 (0.009)	0.034 (0.007)	0.028 (0.006)	0.967 (0.205)	0.967 (0.206)	0.821 (0.194)
MOR (95% CI)	1.41 (1.31-1.50)	1.32 (1.24-1.39)	1.27 (1.20-1.33)	1.22 (1.17-1.26)	1.19 (1.15-1.23)	1.17 (1.13-1.21)	2.54 (2.04-3.04)	2.55 (2.04-3.04)	2.36 (1.88-2.82)

Abbreviation: MOR, median odds ratio.

^a^ Null model.

^b^ Adjusted for age, race/ethnicity, educational level, insurance, prepregnancy body mass index, smoking, prepregnancy diabetes, and previous live birth.

^c^ Adjusted for age, race/ethnicity, educational level, insurance, prepregnancy body mass index, smoking, prepregnancy diabetes, previous live birth, state median income as quintiles, number of general practitioner as quintiles, and percentage of residents whose income in the past 12 months was below the poverty level.

^d^ Adjusted for age, race/ethnicity, educational level, insurance, prepregnancy body mass index, smoking, prepregnancy diabetes, previous live birth, gestational diabetes, plurality, and prenatal care.

^e^ Adjusted for age, race/ethnicity, educational level, insurance, prepregnancy body mass index, smoking, prepregnancy diabetes, previous live birth, gestational diabetes, plurality, prenatal care, state median income as quintiles, number of obstetrician-gynecologist as quintiles, and percentage of residents whose income in the past 12 months was below the poverty level.

### Secondary Analyses

We performed prespecified stratified analyses to examine the crude prevalence of eclampsia for women with and without hypertensive disorders of pregnancy. eFigure 5 and eFigure 6 in the Supplement present caterpillar plots for the unadjusted statewide prevalence of eclampsia among women with and without hypertensive disorders of pregnancy. Among women with hypertensive disorders of pregnancy, 3 states had a high prevalence of eclampsia: Alaska (5.5%), Missouri (5.4%), and Alabama (5.4%). Among women without hypertensive disorders of pregnancy, the prevalence of eclampsia in Hawaii (3.2%) was substantially higher than all other states.

After excluding counties with less than 100 deliveries in 2017, we performed prespecified analyses to examine the crude prevalence of chronic hypertension and hypertensive disorders of pregnancy in 1501 counties and of eclampsia in 1430 counties. eFigures 7, 8, and 9 in the Supplement are heat maps for the unadjusted prevalence in counties of chronic hypertension, hypertensive disorders of pregnancy, and eclampsia.

## Discussion

Our analysis of 3 855 500 US live births in 2017 provides what is, to our knowledge, the most recent prevalence estimates of chronic hypertension, hypertensive disorders of pregnancy, and eclampsia in the US. We observed that the median odds of eclampsia were 2.4-fold higher if the same woman delivered in a US state with a higher vs lower prevalence of eclampsia. There was substantially less state-level variation in the prevalence of chronic hypertension and hypertensive disorders of pregnancy. These data suggest that public health efforts are needed to understand and reduce the degree of statewide variation in the prevalence of eclampsia.

Sparse US data exist on regional variation in the prevalence of hypertensive disorders of pregnancy. A population-based study examining hospital discharge data reported that the prevalence of preeclampsia and eclampsia were highest in the South (3.4%) and Northeast (1.2%).^8^ However, no state-specific prevalence was reported. Our modeled estimates may guide national efforts for hypertension prevention and treatment, especially in states with the highest prevalence of hypertensive disorders of pregnancy and eclampsia.

Hawaii had a very low prevalence of chronic hypertension and hypertensive disorders of pregnancy but the highest prevalence of eclampsia. Possible explanations for these discordant findings are that in women with eclampsia, hypertension may have been underreported or underdiagnosed. A study reporting state-level estimates of hypertension prevalence in US adults between 2013 and 2015 using National Health and Examination Survey data and Behavioral Risk Factor Surveillance System data reported that the prevalence of undiagnosed hypertension was highest in Hawaii (6.5%).^23^ Population-wide studies of blood pressure data are needed to ascertain whether underreporting or underdiagnosis explain why the prevalence of eclampsia was discordant in select states, such as Hawaii.

To our knowledge, this analysis reveals new evidence that variability exists in the prevalence of each hypertensive disorder, especially eclampsia. Patient-level and state-level variables only modestly attenuated the point estimates for the MORs for each disorder. Our post hoc analyses of crude county-level prevalence for each hypertensive disorder did not suggest obvious geographic areas of clustering. Given the substantial statewide variation in eclampsia prevalence and the association of eclampsia with maternal morbidity (eg, disseminated intravascular coagulation, pulmonary edema, cardiac arrest, and perinatal mortality from placental abruption, prematurity, and severe fetal growth restriction),^24^ studies are needed to explore reasons for this variation. Potential state or local measures that may explain this variation include differences in the screening and use of magnesium sulfate in women with impending eclampsia or severe preeclampsia,^25^ state-level economic factors, quality and access to adequate and equitable antenatal and intrapartum care, and distributions of physical or environmental etiologic factors. Detailed analyses of prehospital and hospital-based care in the highest and lowest prevalence counties and states would be an important first step in reducing regional differences in eclampsia. Linkage of existing data sets, such as electronic health records and registries containing blood pressure data with other data on health and nonhealth exposures, may also inform how to improve hypertensive disease surveillance and treatment before and during pregnancy.

### Strengths and Limitations

Strengths of our study include a large sample of approximately 4 million births representing all US live births in 2017, information about the states and counties where delivery occurred, and a large selection of individual-level covariates. This information allowed robust analyses of statewide variation for the 3 hypertensive disorders.

This study has limitations. First, the observational design limits our ability to identify what state attributes influence the prevalence of each disorder. Second, owing to the classification of hypertensive disorders on the birth certificate, we could not ascertain hypertension severity or the timing of the diagnosis. However, in an observational study of preeclampsia and eclampsia in Australia, nearly half (44%) of all eclamptic seizures occurred during labor.^26^ Third, 17% to 25% of women with chronic hypertension develop superimposed preeclampsia^27^; therefore, it is unclear how these diagnoses are coded on the birth certificate. Fourth, coding variability may exist for each hypertensive disorder among states. In a study examining the accuracy of variables on birth certificates in New York City and Vermont, the sensitivity for any hypertensive disorder varied from poor in New York City (39%) to good in Vermont (76%).^14^ However, the specificity (New York City: 99%, Vermont: 99%), positive predictive values (New York City: 86%, Vermont: 95%), and negative predictive values (New York City: 95%, Vermont: 97%) were high. To enable assessment of future changes to hypertension screening and treatment, there is a public health need to assess state-level coding accuracy of hypertension disorders recorded on US birth certificates. Because of either low birth volume or lack of reporting, we could not estimate the degree of county-level variation in the prevalence for each hypertensive disorder. Obtaining accurate data for all US counties may help guide public health officials to narrow the geographic areas most in need of improved surveillance and treatment, especially in high prevalence states. Fifth, because the 95% CIs were relatively wide in states with a high prevalence of eclampsia, there is uncertainty about the accuracy of the prevalence estimates for these states. Nonetheless, the lower bounds of the prevalence estimate in the highest prevalence states for eclampsia (Hawaii, Alaska, Virginia, and Alabama) were above the upper bounds for most other states.

## Conclusions

The findings of this study suggest that substantial differences exist in state-level prevalence of eclampsia within the US. Smaller differences appear to be present for chronic hypertension and hypertensive disorders of pregnancy. These data can inform future public health inquiries to identify reasons for the state-level variability in eclampsia prevalence.

## Notes

Supplement. **eMethods.** Statistical Approach Used in the Random-Effects Models **eReferences** **eTable 1.** Frequencies of Missingness for Each Variable by State **eTable 2.** Adjusted Odds Ratios for Patient Characteristics From the Multilevel Models for Each Hypertensive Disorder **eTable 3.** US States With the 10 Highest and Lowest Adjusted Prevalence of Chronic Hypertension, Hypertensive Disorders of Pregnancy, and Eclampsia in the United States, 2017 **eFigure 1.** Study Flow Chart **eFigure 2.** Heat Map of Adjusted Prevalence of Chronic Hypertension by State **eFigure 3.** Heat Map of Adjusted Prevalence of Hypertensive Disorders of Pregnancy by State **eFigure 4.** Heat Map of Adjusted Prevalence of Eclampsia by State **eFigure 5.** Caterpillar Plot for the Unadjusted Prevalence of Eclampsia Among Women With Hypertensive Disorders of Pregnancy **eFigure 6.** Caterpillar Plot for the Unadjusted Prevalence of Eclampsia Among Women Without Hypertensive Disorders of Pregnancy **eFigure 7.** Heat Map of Crude Prevalence of Chronic Hypertension by County **eFigure 8.** Heat Map of Crude Prevalence of Hypertensive Disorders of Pregnancy by County **eFigure 9.** Heat Map of Crude Prevalence of Eclampsia by County
