# Supplementary material for: Evaluation of US State–Level Variation in Hypertensive Disorders of Pregnancy
Source: JAMA Netw Open. 2020 Oct 1;3(10):e2018741. doi: 10.1001/jamanetworkopen.2020.18741 (PMC7530635; doi:10.1001/jamanetworkopen.2020.18741)
Supplement: Supplement. — eMethods. Statistical Approach Used in the Random-Effects Models eReferences eTable 1. Frequencies of Missingness for Each Variable by State eTable 2. Adjusted Odds Ratios for Patient Characteristics From the Multilevel Models for Each Hypertensive Disorder eTable 3. US States With the 10 Highest and Lowest Adjusted Prevalence of Chronic Hypertension, Hypertensive Disorders of Pregnancy, and Eclampsia in the United States, 2017 eFigure 1. Study Flow Chart eFigure 2. Heat Map of Adjusted Prevalence of Chronic Hypertension by State eFigure 3. Heat Map of Adjusted Prevalence of Hypertensive Disorders of Pregnancy by State eFigure 4. Heat Map of Adjusted Prevalence of Eclampsia by State eFigure 5. Caterpillar Plot for the Unadjusted Prevalence of Eclampsia Among Women With Hypertensive Disorders of Pregnancy eFigure 6. Caterpillar Plot for the Unadjusted Prevalence of Eclampsia Among Women Without Hypertensive Disorders of Pregnancy eFigure 7. Heat Map of Crude Prevalence of Chronic Hypertension by County eFigure 8. Heat Map of Crude Prevalence of Hypertensive Disorders of Pregnancy by County eFigure 9. Heat Map of Crude Prevalence of Eclampsia by County [file jamanetwopen-e2018741-s001.pdf]

Supplementary Online Content

Butwick AJ, Druzin ML, Shaw GM, Guo N. Evaluation of US state–level variation in hypertensive disorders of pregnancy. *JAMA Netw Open*. 2020;3(10):e2018741. doi:10.1001/jamanetworkopen.2020.18741

eMethods. Statistical Approach Used in the Random-Effects Models

eReferences

eTable 1. Frequencies of Missingness for Each Variable by State

eTable 2. Adjusted Odds Ratios for Patient Characteristics From the Multilevel Models for Each Hypertensive Disorder

eTable 3. US States With the 10 Highest and Lowest Adjusted Prevalence of Chronic Hypertension, Hypertensive Disorders of Pregnancy and Eclampsia in the United States, 2017

eFigure 1. Study Flow Chart

eFigure 2. Heat Map of Adjusted Prevalence of Chronic Hypertension by State

eFigure 3. Heat Map of Adjusted Prevalence of Hypertensive Disorders of Pregnancy by State

eFigure 4. Heat Map of Adjusted Prevalence of Eclampsia by State

eFigure 5. Caterpillar Plot for the Unadjusted Prevalence of Eclampsia Among Women With Hypertensive Disorders of Pregnancy

eFigure 6. Caterpillar Plot for the Unadjusted Prevalence of Eclampsia Among Women Without Hypertensive Disorders of Pregnancy

eFigure 7. Heat Map of Crude Prevalence of Chronic Hypertension by County

eFigure 8. Heat Map of Crude Prevalence of Hypertensive Disorders of Pregnancy by County

eFigure 9. Heat Map of Crude Prevalence of Eclampsia by County

This supplementary material has been provided by the authors to give readers additional information about their work.

© 2020 Butwick AJ et al. *JAMA Network Open.*

eMethods: Statistical Approach used in the random-effects models.

Multilevel logistic regression was fit by the GLMIMIX procedure in SAS 9.4 (SAS Institute Inc, Cary, NC) using maximum likelihood estimation based on Laplace approximation. Three regression models were fit sequentially: First, we developed an unconditional or 'null' model including the state identifier as a random effect was fitted. In the null model, the random effect may be interpreted as each state's deviation from the mean state-level hypertensive disorders prevalences. In the second model, patient-level factors were added to determine how much of the variation can be explained by patient-level factors. In the third model, 2017 state median income and percent of families with a household income below the poverty level were added, whilst controlling for individual-level determinants of hypertensive disorders. For the model with chronic hypertension, we also included data for the number of general practitioners per 1000 deliveries. For the models for hypertensive disorders of pregnancy and eclampsia, we also included data for the number of obstetricians-gynecologists per 1000 deliveries. Because of a non-linear relationship between state median income and number of medical practitioners with relevant outcomes and for greater ease of interpretation of rates, we categorized state median income and number of medical practitioners by quintiles. The adjusted state-specific prevalence of hypertensive disorders can be interpreted as each state's deviation from the mean hypertensive disorders prevalences that is not accounted for by covariates. For each model, the state-level hypertensive disorders prevalences and 95% confidence interval (CI) were calculated by first converting each state's estimated odds ratio for hypertensive disorders obtained from the multilevel logistic regression model (exponential of random effect) into a relative risk and then multiplying by the national rate.[1] The association between individual patient-level variables and hypertensive disorders was measured by the regression coefficients and 95% CI.

In order to quantify and interpret the variance across states, we calculated the median odds ratio (MOR). The MOR estimates the odds of hypertensive disorders that would occur if a patient moved from a state with a low odds of hypertensive disorders to a state with a high odds of hypertensive disorders, after case-mix adjustment.[1,2] The MOR is computed by translating the state-level variance into an odds scale. $MOR = \exp\,[\sqrt{2 \times V_A} \times 0.6745]$ (V_A = area residual variance on the logistic scale).[2] In addition, we compared the MORs across models (null model vs. adjusted model) to examine the extent to which variables explain variance in the odds of the intervention across states. If the MOR=1, then no variation exists among states; if the MOR>1, variation is present among states, with the variation increasing as the MOR increases. To obtain 95% confidence intervals for the MOR, we applied the formula to the upper and lower 95% confidence limits of the random-effects variance estimated by profile-likelihood.[3,4]

© 2020 Butwick AJ et al. *JAMA Network Open.*

eReferences

1. Larsen K, Petersen JH, Budtz-Jorgensen E, Endahl L. Interpreting parameters in the logistic regression model with random effects. Biometrics 2000;56:909-14.
2. Merlo J, Chaix B, Ohlsson H, Beckman A, Johnell K, Hjerpe P, Rastam L, Larsen K. A brief conceptual tutorial of multilevel analysis in social epidemiology: using measures of clustering in multilevel logistic regression to investigate contextual phenomena. J Epidemiol Community Health 2006;60:290-7.
3. Saha KK. Profile likelihood-based confidence interval of the intraclass correlation for binary outcome data sampled from clusters. Stat Med 2012; 31: 3982-4002.
4. Zou G, Donner A. Confidence interval estimation of the intraclass correlation coefficient for binary outcome data. Biometrics 2004; 60: 807-11.

© 2020 Butwick AJ et al. *JAMA Network Open.*

eTable 1. Frequencies of missingness for each variable by state

State	cHTN	HDP	Ecl	Age	Race	Edu	Ins	Smoke	BMI	GDM	PDM	PLB	Plural	Prenatal care	Total
**AK**	1.00	1.00	1.00	0.00	1.17	1.45	1.71	2.22	2.42	1.00	1.00	0.33	0.00	2.14	10.56
**AL**	0.00	0.00	0.00	0.00	0.02	0.25	0.05	0.42	1.97	0.00	0.00	0.00	0.00	0.43	2.87
**AR**	0.00	0.00	0.00	0.00	0.42	0.54	1.43	2.31	7.13	0.00	0.00	2.26	0.00	5.59	14.82
**AZ**	0.00	0.00	0.00	0.00	0.58	0.68	0.20	0.23	0.27	0.00	0.00	0.06	0.00	2.25	3.70
**CA**	0.00	0.00	0.00	0.00	2.89	5.06	0.11	0.55	2.53	0.00	0.00	0.06	0.00	1.19	8.91
**CO**	0.01	0.01	0.01	0.00	1.50	1.38	0.31	0.37	4.34	0.01	0.01	0.11	0.00	1.79	8.05
**CT**	0.76	0.76	0.76	0.00	0.09	0.27	0.19	0.12	0.45	0.76	0.76	0.13	0.00	2.58	3.61
**DC**	0.00	0.00	0.00	0.00	0.00	0.44	0.25	0.50	2.73	0.00	0.00	0.01	0.00	2.69	5.71
**DE**	0.01	0.01	0.01	0.00	0.09	0.44	0.04	0.05	1.37	0.01	0.01	0.01	0.00	1.55	3.20
**FL**	0.23	0.23	0.23	0.00	0.27	1.08	0.42	0.81	6.48	0.23	0.23	0.39	0.00	6.11	12.66
**GA**	0.30	0.30	0.30	0.00	0.95	0.63	0.18	0.20	1.39	0.30	0.30	0.33	0.00	1.51	4.51
**HI**	0.00	0.00	0.00	0.00	0.30	0.65	0.15	1.58	4.83	0.00	0.00	0.00	0.00	6.70	10.15
**IA**	0.00	0.00	0.00	0.00	0.01	0.16	0.01	0.04	0.23	0.00	0.00	0.01	0.00	0.25	0.66
**ID**	0.07	0.07	0.07	0.00	0.31	0.50	0.05	0.17	0.96	0.07	0.07	0.11	0.00	0.43	2.25
**IL**	0.25	0.25	0.25	0.00	1.09	2.24	0.43	0.36	7.19	0.25	0.25	0.29	0.00	4.33	11.50
**IN**	0.01	0.01	0.01	0.00	0.02	0.14	0.20	0.07	0.45	0.01	0.01	0.03	0.00	0.14	0.94
**KS**	0.00	0.00	0.00	0.00	0.11	0.31	0.31	0.23	0.30	0.00	0.00	0.01	0.00	0.14	1.29
**KY**	0.39	0.39	0.39	0.00	0.27	0.47	0.78	0.86	0.92	0.39	0.39	0.00	0.00	4.72	7.47
**LA**	0.00	0.00	0.00	0.00	0.05	0.68	0.00	0.85	2.25	0.00	0.00	0.08	0.00	2.51	5.80
**MA**	0.10	0.10	0.10	0.00	2.52	3.83	0.15	0.01	2.11	0.10	0.10	0.22	0.00	1.21	6.45
**MD**	0.10	0.10	0.10	0.00	0.29	0.57	0.53	0.42	3.54	0.10	0.10	0.23	0.00	4.84	9.34
**ME**	0.00	0.00	0.00	0.00	0.00	0.15	0.32	0.02	0.30	0.00	0.00	0.07	0.00	0.17	0.91
**MI**	0.28	0.28	0.28	0.00	0.19	1.04	0.29	0.84	2.98	0.28	0.28	0.07	0.00	3.06	6.94
**MN**	0.17	0.17	0.17	0.00	0.55	1.13	0.20	0.79	2.28	0.17	0.17	0.19	0.00	0.92	4.77
**MO**	0.00	0.00	0.00	0.00	0.46	0.36	1.46	1.00	1.44	0.00	0.00	0.47	0.00	6.46	10.31
**MS**	0.03	0.03	0.03	0.00	0.02	0.19	0.06	0.17	0.31	0.03	0.03	0.01	0.00	0.53	1.13
**MT**	0.06	0.06	0.06	0.00	0.29	0.39	0.20	0.62	0.76	0.06	0.06	0.03	0.00	0.26	2.02
**NC**	0.01	0.01	0.01	0.00	0.08	0.21	0.08	0.01	2.14	0.01	0.01	0.02	0.00	0.61	3.01
**ND**	0.00	0.00	0.00	0.00	1.67	1.33	1.28	0.94	0.92	0.00	0.00	0.04	0.00	5.07	9.38
**NE**	0.11	0.11	0.11	0.00	0.06	0.04	1.29	0.08	0.97	0.11	0.11	0.19	0.00	2.70	5.18
**NH**	0.41	0.41	0.41	0.00	0.44	0.35	0.70	0.82	3.57	0.41	0.41	0.22	0.00	0.23	6.09
**NJ**	0.00	0.00	0.00	0.00	1.47	0.68	0.00	0.52	0.54	0.00	0.00	0.03	0.00	1.51	4.25
**NM**	0.00	0.00	0.00	0.00	0.25	0.22	0.12	0.45	1.40	0.00	0.00	0.37	0.00	2.47	4.51
**NV**	0.00	0.00	0.00	0.00	0.47	2.95	0.65	0.85	3.03	0.00	0.00	0.16	0.00	5.99	11.68
**NY**	0.18	0.18	0.18	0.00	0.91	0.52	0.38	0.09	2.89	0.18	0.18	0.58	0.00	2.35	5.72

© 2020 Butwick AJ et al. *JAMA Network Open.*

**OH**	0.00	0.00	0.00	0.00	0.16	0.28	0.68	0.15	1.01	0.00	0.00	0.28	0.00	1.11	3.22
**OK**	0.00	0.00	0.00	0.00	0.14	0.26	0.23	0.16	0.71	0.00	0.00	0.14	0.00	2.38	3.40
**OR**	0.00	0.00	0.00	0.00	0.73	0.48	0.23	0.43	0.93	0.00	0.00	0.15	0.00	0.72	3.22
**PA**	0.00	0.00	0.00	0.00	1.00	0.70	3.33	1.27	6.68	0.00	0.00	0.49	0.00	2.66	14.42
**RI**	0.03	0.03	0.03	0.00	0.80	2.70	0.09	2.09	5.85	0.03	0.03	2.20	0.00	4.89	13.51
**SC**	0.00	0.00	0.00	0.00	0.21	0.31	0.28	0.09	1.33	0.00	0.00	0.04	0.00	0.13	2.14
**SD**	0.04	0.04	0.04	0.00	0.05	0.34	0.42	0.29	0.85	0.04	0.04	0.05	0.00	1.17	2.86
**TN**	0.07	0.07	0.07	0.00	0.11	0.64	4.99	0.73	2.78	0.07	0.07	1.07	0.00	6.82	13.96
**TX**	0.00	0.00	0.00	0.00	0.09	0.15	0.19	0.02	0.40	0.00	0.00	0.04	0.00	1.49	2.12
**UT**	0.00	0.00	0.00	0.00	0.95	1.65	3.34	0.32	1.48	0.00	0.00	0.04	0.00	1.37	7.31
**VA**	0.00	0.00	0.00	0.00	0.19	1.03	0.15	1.18	2.60	0.00	0.00	0.06	0.00	6.35	10.18
**VT**	0.00	0.00	0.00	0.00	0.94	0.63	0.29	1.41	1.27	0.00	0.00	0.09	0.00	0.42	4.68
**WA**	0.43	0.43	0.43	0.00	2.63	1.51	1.62	1.35	4.14	0.43	0.43	1.11	0.00	5.91	14.07
**WI**	0.27	0.27	0.27	0.00	0.46	0.49	1.35	0.68	1.92	0.27	0.27	0.22	0.00	3.16	7.54
**WV**	0.55	0.55	0.55	0.00	0.67	0.45	0.60	0.52	0.63	0.55	0.55	0.66	0.00	0.79	4.08
**WY**	0.00	0.00	0.00	0.00	1.85	0.75	0.32	2.61	0.61	0.00	0.00	0.02	0.00	0.96	5.93
**All**	0.10	0.10	0.10	0.00	0.84	1.29	0.61	0.49	2.52	0.10	0.10	0.25	0.00	2.55	6.89

cHTN = chronic hypertension; HDP = hypertensive disorders of pregnancy; Ecl = eclampsia; Edu = Education; Ins = insurance; BMI = body mass index; GDM = gestational diabetes; PDM = prepregnancy diabetes; PLB = prior live birth

© 2020 Butwick AJ et al. *JAMA Network Open.*

eTable 2. Adjusted models for chronic hypertension, hypertensive disorders of pregnancy, and eclampsia in women delivered a live birth in US 2017

Variables	Chronic hypertension[a] aOR (95% CI)	Hypertensive disorders of pregnancy[b] aOR (95% CI)	Eclampsia[b] aOR (95% CI)
**Maternal age (years)**			
**25-29**	Ref	Ref	Ref
**<20 years**	0.47 (0.45, 0.50)	1.02 (0.99, 1.04)	1.08 (0.99, 1.19)
**20-24**	0.67 (0.65, 0.69)	0.97 (0.96, 0.98)	0.97 (0.92, 1.03)
**30-34**	1.56 (1.53, 1.59)	1.08 (1.06, 1.09)	1.12 (1.06, 1.19)
**35-39**	2.39 (2.34, 2.45)	1.23 (1.22, 1.25)	1.31 (1.23, 1.40)
**≥40**	3.73 (3.61, 3.85)	1.54 (1.51, 1.58)	1.76 (1.59, 1.96)
**Maternal race/ethnicity**			
**Non-Hispanic White**	Ref	Ref	Ref
**Non-Hispanic Black**	1.79 (1.75, 1.82)	1.06 (1.05, 1.07)	1.37 (1.29, 1.45)
**Non-Hispanic Other**	1.14 (1.09, 1.19)	0.98 (0.95, 1.00)	1.10 (1.00, 1.21)
**Non-Hispanic Asian**	0.87 (0.83, 0.90)	0.65 (0.63, 0.66)	0.91 (0.83, 1.01)
**Hispanic**	0.77 (0.76, 0.79)	0.81 (0.80, 0.82)	1.03 (0.97, 1.10)
**Insurance**			
**Private insurance**	Ref	Ref	Ref
**Medicaid**	1.10 (1.08, 1.12)	0.96 (0.95, 0.97)	1.07 (1.02, 1.13)
**Selfpay**	0.71 (0.67, 0.75)	0.71 (0.69, 0.73)	0.95 (0.84, 1.07)
**Other**	1.30 (1.25, 1.36)	0.94 (0.92, 0.96)	1.20 (1.09, 1.33)
**Maternal education**			
**High school and under**	Ref	Ref	Ref
**College/associated/bachelor**	0.94 (0.92, 0.96)	1.00 (0.99, 1.01)	0.92 (0.88, 0.97)
**Master/doctorate**	0.80 (0.77, 0.82)	0.90 (0.88, 0.92)	0.76 (0.70, 0.82)
**Pre-pregnancy smoking**	1.26 (1.23, 1.29)	1.00 (0.98, 1.02)	0.92 (0.86, 0.99)
**Pre-pregnancy BMI (kg/m^2)**			
**Normal or underweight**	Ref	Ref	Ref
**Overweight**	2.04 (1.99, 2.09)	1.67 (1.65, 1.69)	1.47 (1.39, 1.55)
**Obesity class I**	3.58 (3.50, 3.67)	2.33 (2.30, 2.36)	1.95 (1.85, 2.07)
**Obesity class II**	5.56 (5.41, 5.71)	2.96 (2.91, 3.00)	2.37 (2.21, 2.54)
**Obesity class III**	10.06 (9.81, 10.32)	3.87 (3.81, 3.94)	2.80 (2.61, 3.01)
**Pre-pregnancy diabetes**	5.38 (5.20, 5.55)	2.05 (1.99, 2.12)	2.62 (2.33, 2.95)
**Gestational diabetes**	-	1.72 (1.70, 1.75)	1.56 (1.46, 1.67)

© 2020 Butwick AJ et al. *JAMA Network Open.*

**Prior livebirth**			
**No prior live birth**	Ref	Ref	Ref
**1 or more prior live birth**	0.79 (0.78, 0.81)	0.50 (0.50, 0.51)	0.53 (0.51, 0.56)
**Plurality**			
**Singleton pregnancy**	-	Ref	Ref
**Twin pregnancy**	-	2.22 (2.18, 2.26)	2.61 (2.43, 2.82)
**Triplet or higher order pregnancy**	-	2.65 (2.41, 2.91)	3.74 (2.67, 5.25)
**Trimester prenatal care initiated**	-		
**1st trimester**	-	Ref	Ref
**2nd trimester**	-	0.94 (0.93, 0.95)	1.00 (0.95, 1.06)
**3rd trimester**	-	0.88 (0.86, 0.90)	0.90 (0.81, 1.00)
**No prenatal care**	-	0.92 (0.89, 0.96)	1.63 (1.41, 1.88)

[a] Adjusted for maternal age, race/ethnicity, education, insurance, prepregnancy body mass index (BMI) using the World Health Organization BMI categories, smoking history before pregnancy, prepregancy diabetes, number of prior livebirths.

[b] Adjusted for maternal age, race/ethnicity, education, insurance, prepregnancy body mass index (BMI) using the World Health Organization BMI categories, smoking history before pregnancy, prepregancy diabetes, number of prior livebirths, plurality, gestational diabetes.

[c] South Carolina and Tennessee were excluded since they did not report eclampsia

aOR = Adjusted odds ratio; CI = confidence intervals; BMI = body mass index

© 2020 Butwick AJ et al. *JAMA Network Open.*

eTable 3. US states with the 10 highest and lowest adjusted prevalence of chronic hypertension, hypertensive disorders of pregnancy and, eclampsia in the United States, 2017

	Chronic hypertension				Hypertensive disorders of pregnancy				Eclampsia			
	Top 10 Highest Adjusted Prevalance		Top 10 Lowest Adjusted Prevalence		Top 10 Highest Adjusted Prevalence		Top 10 Lowest Adjusted Prevalence		Top 10 Highest Adjusted Prevalence		Top 10 Lowest Adjusted Prevalence	
	State	% (95% CI)	State	% (95% CI)	State	% (95% CI)	State	% (95% CI)	State	% (95% CI)	State	% (95% CI)
1	AK	3.39 (2.98-3.86)	HI	1.04 (0.88-1.23)	LA	9.30 (8.85-9.77)	MA	4.34 (4.08-4.61)	HI	2.76 (2.22-3.36)	DE	0.03 (0.01-0.09)
2	TN	3.02 (2.77-3.29)	MN	1.09 (0.98-1.21)	NH	8.85 (8.26-9.48)	HI	4.48 (4.10-4.90)	AK	1.76 (1.31-2.35)	UT	0.08 (0.05-0.13)
3	LA	3.01 (2.76-3.29)	CA	1.13 (1.03-1.23)	AK	8.75 (8.11-9.44)	NJ	4.61 (4.35-4.88)	VA	1.60 (1.23-2.07)	ID	0.08 (0.05-0.15)
4	OH	3.00 (2.76-3.25)	WY	1.27 (0.99-1.62)	OH	7.98 (7.61-8.38)	VA	4.67 (4.41-4.94)	AL	1.52 (1.16-1.97)	TX	0.09 (0.07-1.12)
5	DE	2.95 (2.61-3.34)	MT	1.28 (1.07-1.53)	OR	7.82 (7.40-8.27)	CA	4.74 (4.51-4.99)	WY	0.88 (0.58-1.33)	NH	0.09 (0.05-0.18)
6	WV	2.91 (2.60-3.26)	ID	1.30 (1.12-1.51)	VT	7.74 (7.04-8.51)	NV	4.93 (4.59-5.28)	MO	0.87 (0.65-1.16)	MI	0.10 (0.07-0.14)
7	MO	2.72 (2.48-2.97)	MI	1.41 (1.29-1.55)	MO	7.67 (7.28-8.08)	TN	5.23 (4.94-5.54)	OK	0.80 (0.60-1.08)	RI	0.10 (0.05-0.21)
8	MD	2.56 (2.34-2.79)	NV	1.43 (1.26-1.61)	KY	7.58 (7.18-8.00)	AR	5.45 (5.10-5.82)	PA	0.78 (0.59-1.04)	WV	0.11 (0.06-0.18)
9	OK	2.53 (2.30-2.78)	SD	1.44 (1.22-1.70)	SC	7.37 (6.98-7.73)	MS	5.66 (5.32-6.02)	SD	0.77 (0.54-1.10)	CA	0.11 (0.08-0.14)
10	SC	2.52 (2.30-2.76)	AZ	1.46 (1.32-1.61)	UT	7.30 (6.89-7.73)	NY	5.69 (5.41-5.99)	AR	0.66 (0.48-0.90)	FL	0.11 (0.08-0.15)

© 2020 Butwick AJ et al. *JAMA Network Open.*

eFigure 1. Study flow chart

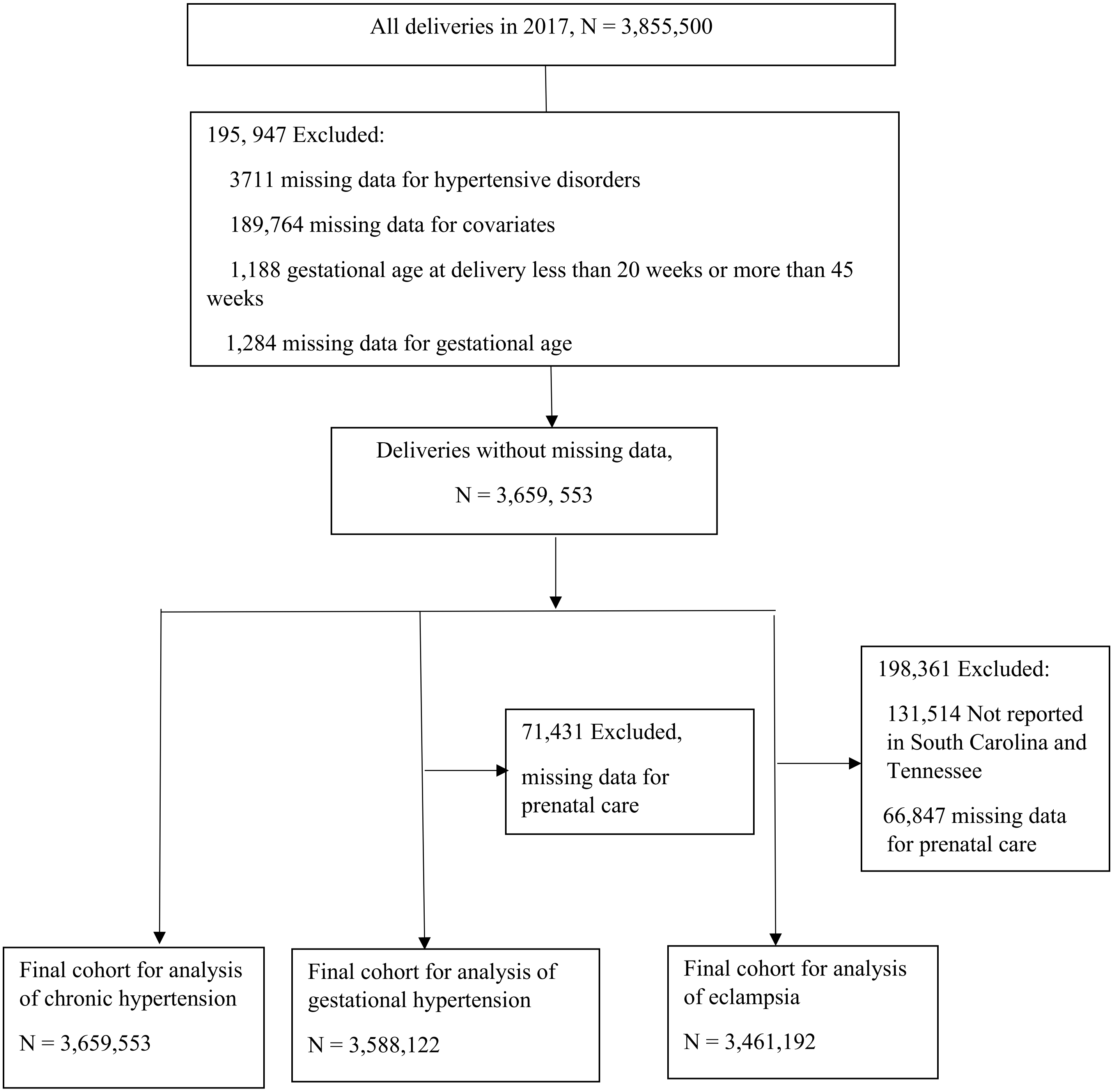

© 2020 Butwick AJ et al. *JAMA Network Open.*

eFigure 2. Heat map of adjusted prevalence of chronic hypertension by state.

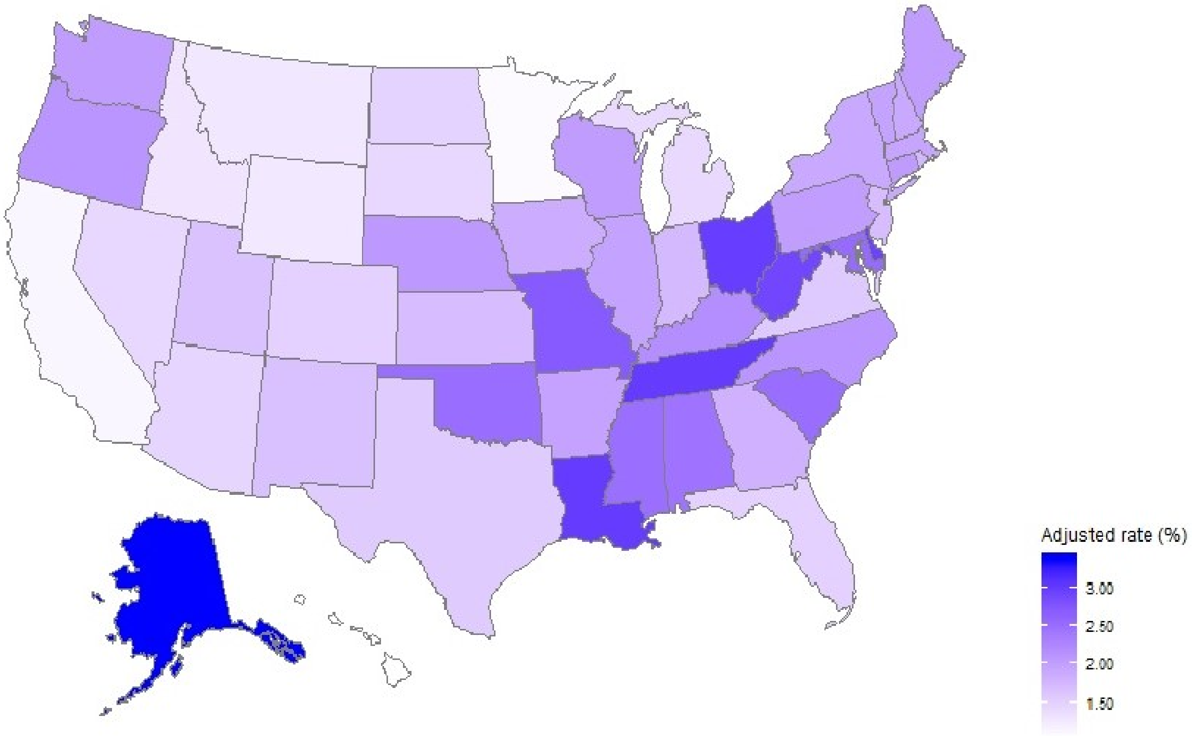

© 2020 Butwick AJ et al. *JAMA Network Open.*

eFigure 3. Heat map of adjusted prevalence of hypertensive disorders of pregnancy by state.

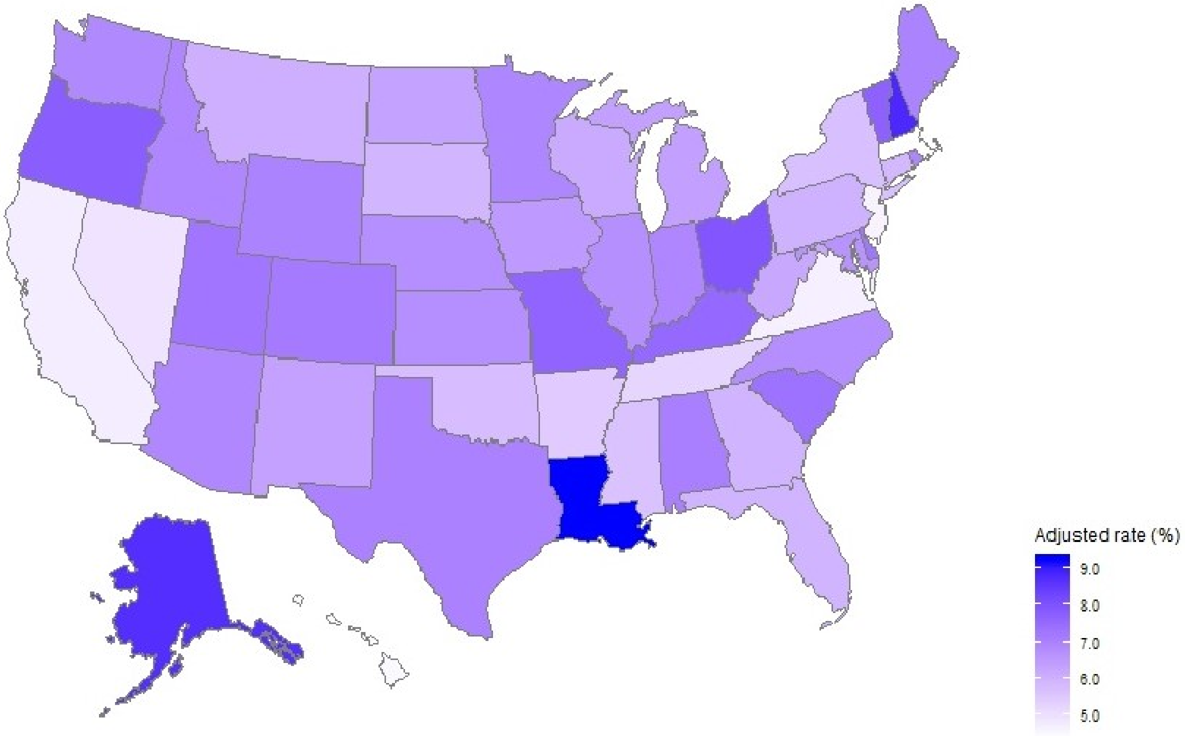

© 2020 Butwick AJ et al. *JAMA Network Open.*

eFigure 4. Heat map of adjusted prevalence of eclampsia by state.

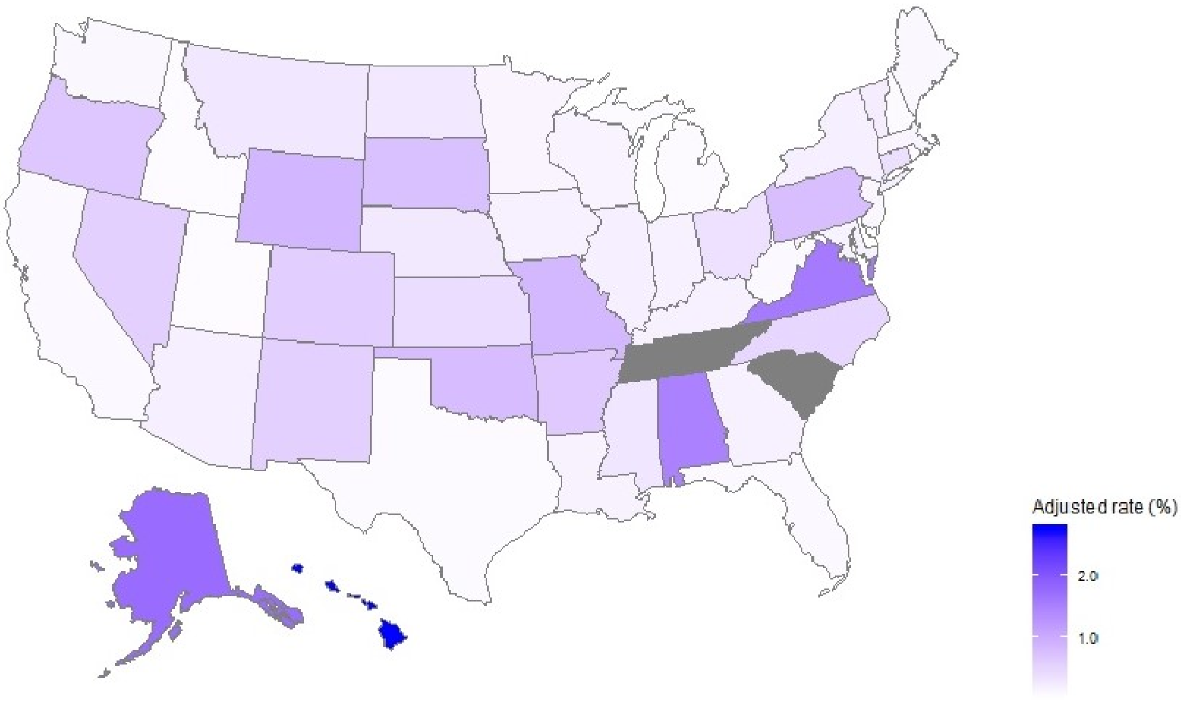

South Carolina and Tennessee did not provide eclampsia data.

© 2020 Butwick AJ et al. *JAMA Network Open.*

eFigure 5. Caterpillar plot for the unadjusted prevalence of eclampsia among women with hypertensive disorders of pregnancy.

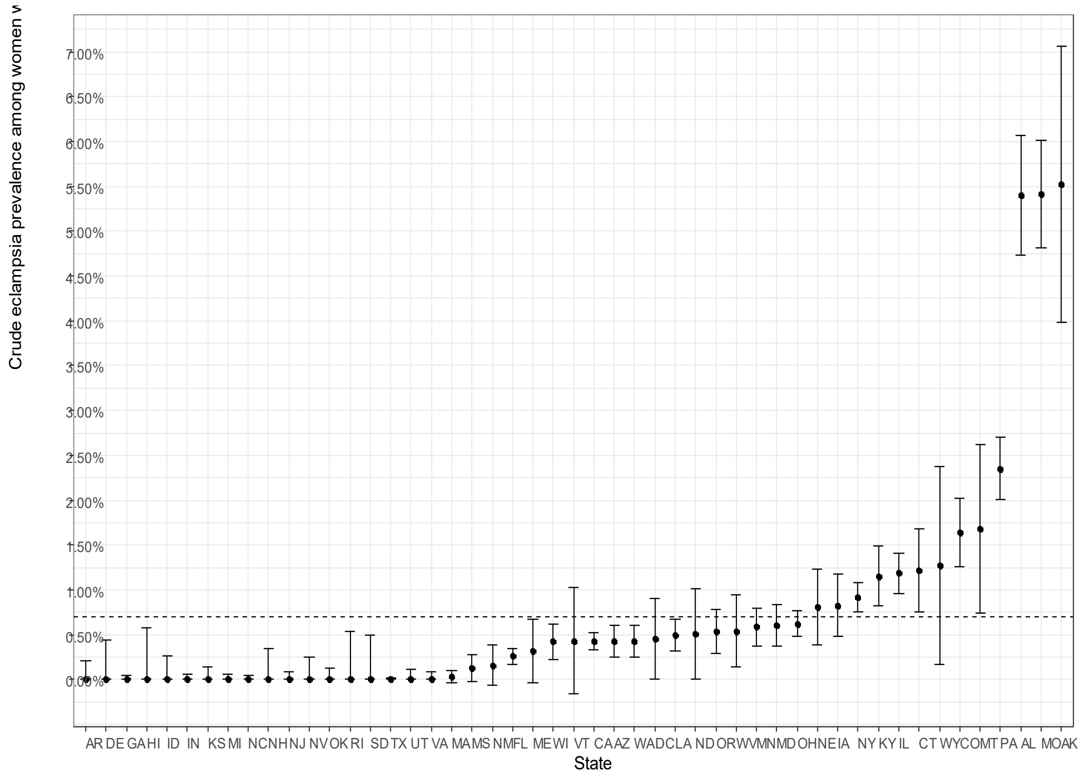

The circles refer to the mean prevalence. The whisker bars indicated 95% CI. Dashed horizontal line refers to the overall mean prevalence for all US states. South Carolina and Tennessee did not provide eclampsia data.

© 2020 Butwick AJ et al. *JAMA Network Open.*

eFigure 6. Caterpillar plot for the unadjusted prevalence of eclampsia among women without hypertensive disorders of pregnancy.

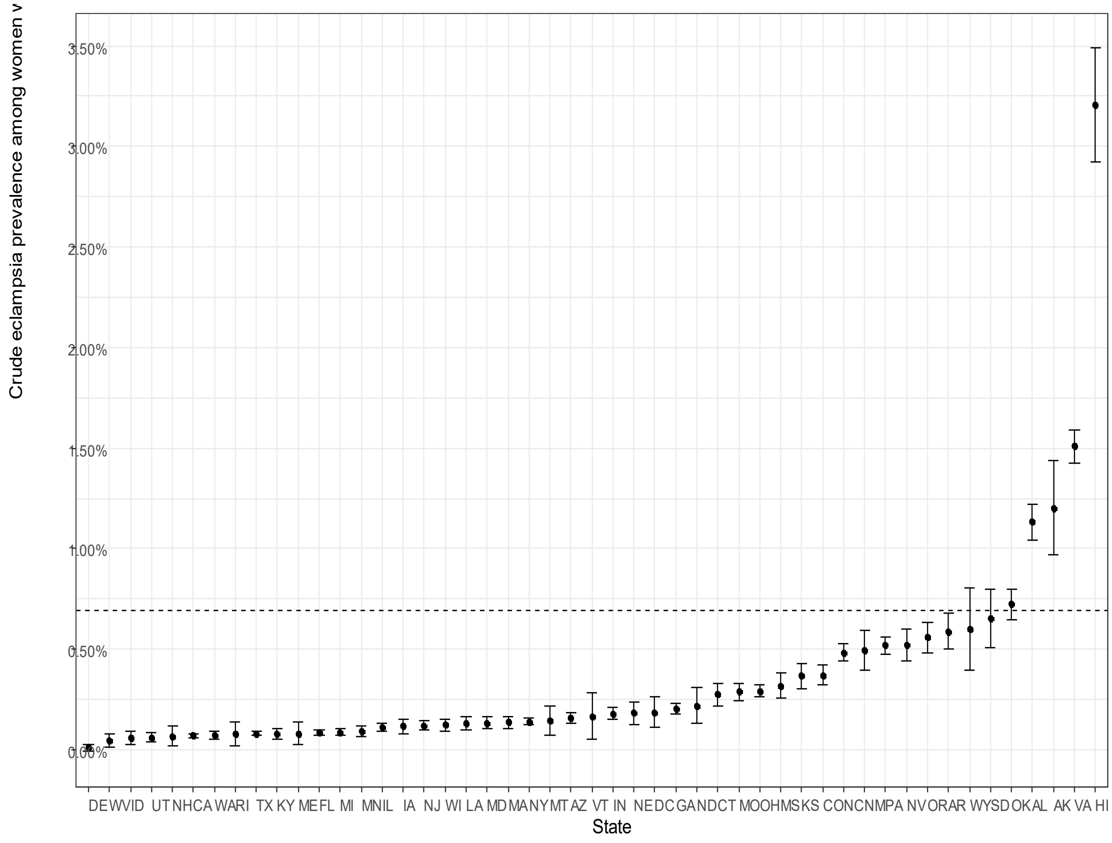

The circles refer to the mean prevalence. The whisker bars indicated 95% CI. Dashed horizontal line refers to the overall mean prevalence for all US states. South Carolina and Tennessee did not provide eclampsia data.

© 2020 Butwick AJ et al. *JAMA Network Open.*

eFigure 7. Heat map of crude prevalence of chronic hypertension by county.

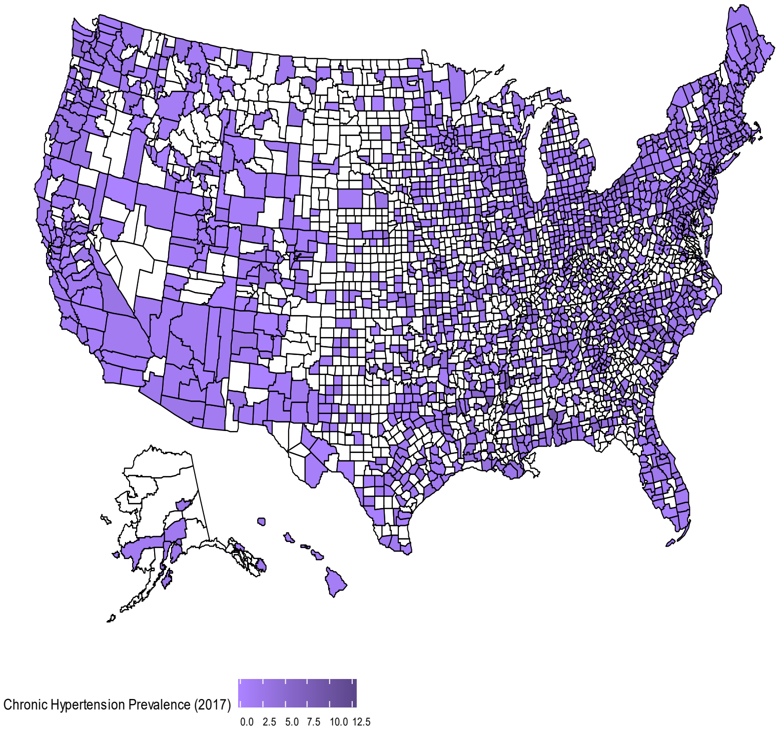

Counties in white are counties that had less than 100 deliveries in 2017 or counties that did not report delivery data.

© 2020 Butwick AJ et al. *JAMA Network Open.*

eFigure 8. Heat map of crude prevalence of hypertensive disorders of pregnancy by county.

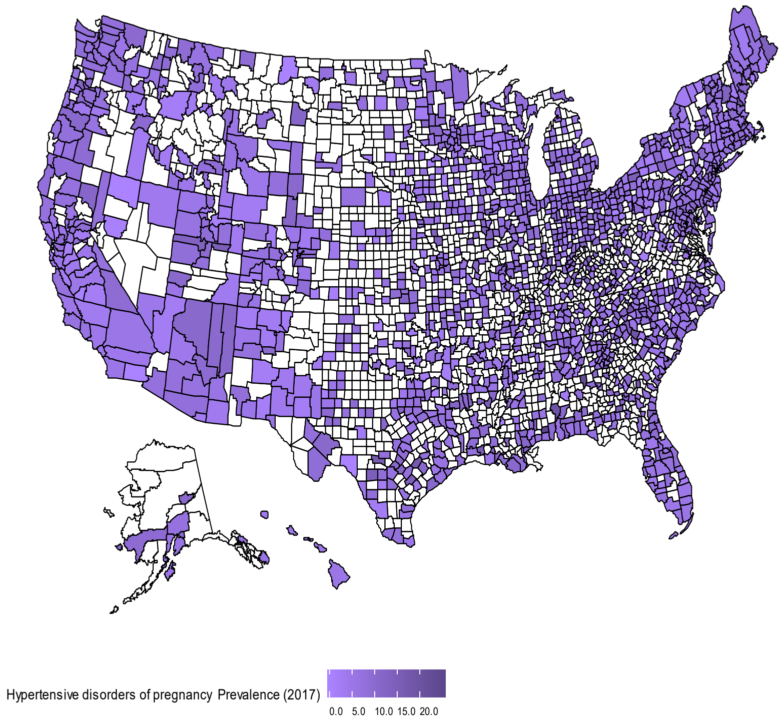

Counties in white are counties that had less than 100 deliveries in 2017 or counties that did not report delivery data.

© 2020 Butwick AJ et al. *JAMA Network Open.*

eFigure 9. Heat map of crude prevalence of eclampsia by county.

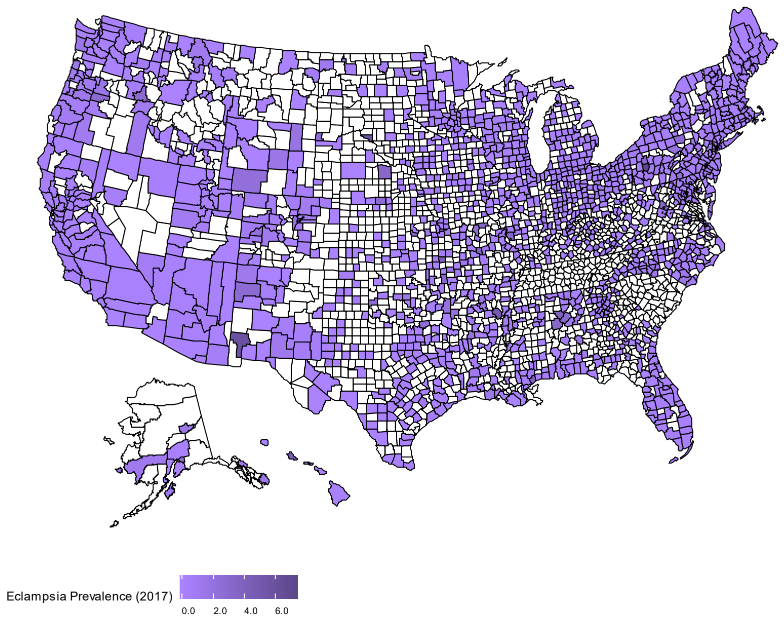

Counties in white are counties that had less than 100 deliveries in 2017 or counties that did not report delivery data. South Carolina and Tennessee did not provide eclampsia data.

© 2020 Butwick AJ et al. *JAMA Network Open.*
